# The relationships between thyroid functions of short-term rapid hypothyroidism and blood lipid levels in post-thyroidectomy patients of differentiated thyroid cancer

**DOI:** 10.3389/fendo.2023.1114344

**Published:** 2023-04-27

**Authors:** Jinming Yao, Junyu Zhao, Jing Liu, Shan Jiang, Siyi Guo, Lusi Xu, Xinzhong Zhang, Qiqi Sheng, Kaili Wang, Lin Liao, Jianjun Dong

**Affiliations:** ^1^ Shandong Key Laboratory of Rheumatic Disease and Translational Medicine, Department of Endocrinology and Metabology, The First Affiliated Hospital of Shandong First Medical University & Shandong Provincial Qianfoshan Hospital, Shandong Institute of Nephrology, Ji-nan, China; ^2^ Department of Endocrinology and Metabology, Shandong Provincial Qianfoshan Hospital, Cheeloo College of Medicine, Shandong University, Ji-nan, China; ^3^ Department of Endocrinology, Shaoguan First Peoples Hospital, Shaoguan, Guangdong, China; ^4^ Department of Endocrinology and Metabology, Qilu Hospital of Shandong University, Cheeloo College of Medicine, Shandong University, Ji-nan, China

**Keywords:** differentiated thyroid cancer, thyroid stimulating hormone, total cholesterol, triglycerides, high density lipoprotein-cholesterol

## Abstract

**Objective:**

To explore the relationship between short-term rapid hypothyroidism and blood lipid levels in patients with differentiated thyroid cancer (DTC).

**Methods:**

Seventy-five DTC patients scheduled to receive radioactive iodine ablation were enrolled. Levels of thyroid hormone and serum lipids were tested at two time points: the euthyroid before thyroidectomy, and the hypothyroid (off thyroxine). Then the collected data were analyzed.

**Results:**

Totally 75 DTC patients enrolled, among them, 5o were female (66.67%) and 25 were male (33. 33%), with an average age of 52.24 ± 1.24 years old. The short-term rapid severe hypothyroidism induced by thyroid hormone withdrawal significantly aggravated dyslipidemia, particularly in patients with dyslipidemia before thyroidectomy (All *P* < 0.01). However, there was no significant differences between blood lipid levels with different thyroid stimulating hormone (TSH) levels. And our study showed significant negative correlations between free triiodothyronine levels and the changes from euthyjroidism to hypothyroidism in total cholesterol (r=-0.31, *P*=0.03), triglycerides (r=-0.39, *P*=0.006), high density lipoprotein-cholesterol (HDL-C) (r=-0.29, *P*=0.042), and significant positive correlations between free thyroxine and the changes of HDL-C (r=-0.32, *P*=0.027) were identified in females, however, which were not observed in males.

**Conclusion:**

Short-term rapids severe hypothyroidism caused by thyroid hormone withdrawal can lead to rapid significant changes in blood lipid levels. It is necessary to pay attention to dyslipidemia and its long-term effects after thyroid hormone withdrawal, especially in patients with dyslipidemia before thyroidectomy.

**Clinical trial registration:**

https://clinicaltrials.gov/ct2/show/NCT03006289?term=NCT03006289&draw=2&rank=1, identifier NCT03006289.

## 1 Introduction

Hypothyroidism is known to have adverse effects on lipoprotein metabolism (1, 2). The levels of serum total cholesterol (TC), low density lipoprotein cholesterol (LDL-C) and triglycerides (TG) in patients with hypothyroidism were significantly higher than those in healthy people (3–5).

Most of previous studies suggested significant associations between chronic hypothyroidism and hyperlipidemia (2, 6, 7). However, most of them are chronic hypothyroidism caused by autoimmune factors.Thyroid stimulating hormone (THW) in DTC patients who have undergone thyroidectomy represents a model of acute overt hypothyroidism. Nearly all DTC patients who scheduled for radioiodine therapy develop a rapid hypothyroidism within about 10 to 14 days. However, few studies have reported the relationship between thyroid function of short-term rapid hypothyroidism and change of blood lipids in DTC patients with THW. Therefore, our study aimed to investigate the relationship between thyroid function and change in blood lipids in DTC patients with THW.

## 2 Materials and methods

### 2.1 Study participants

This study was conducted at Qilu Hospital of Shandong University and Shandong provincial Qianfoshan Hospital. Totally 75 DTC patients with total thyroidectomy, had developed hypothyroidism after THW in order to receive radiodine therapy, were enrolled.

We excluded those with coronary heart, chronic liver and chronic renal diseases, uncontrolled diabetes mellitus and those who had taken lipid-lowering drugs or received other treatments that affect blood lipid levels. The study has been registered in the ClinicalTrial.gov registry (NCT03006289).

### 2.2 Data and measurements

The levels of thyroid hormone and serum lipid were measured at two time points: before thyroidectomy when patients were euthyroid and hypothyroidism after the patients withdrew thyroxine. Fasting venous blood was taken for serum free triiodothyronine (FT3), free thyroxine (FT4), TSH, TC, TG, LDL-C, high density lipoprotein-cholesterol (HDL-C), uric acid (UA) and fasting blood glucose (FBG).

### 2.3 Statistical analyses

Continuous variables were presented as mean ± standard deviation (SD) or median (interquartile range). Categorical variables were presented as frequencies and percentages. The paired sample t-test was used to compare the parameters of DTC patients at the two time points. Pearson correlation analysis and Spearman correlation analysis were used to calculate the correlations between TC, TG, LDL-C, HDL-C and FT3, FT4 and TSH, respectively. χ^2^-test was used to compare categorical variables. All tests were two-sided, and *P*<0.05 was considered statistically significant. All statistical analyses were performed with SPSS 26.0 (IBM SPSS, Armonk, NY, USA).

## 3 Results

### 3.1 General characteristics

A total of 75 patients enrolled this study, including 50 females and 25 males. General characteristics are shown in **Table 1**. The average age of patients was 52.24 (SD=1.24) years old, and the BMI was 25.94 (SD=0.45) kg/m^2^.

**Table 1:** Baseline characteristics of patients with DTC who were scheduled to receive radioactive iodine therapy following thyroidectomy.

Parameters	Patient cohort (n=75)
Gender(male/female)	25/50
Age (years)	52.24 ± 1.24
Height (cm)	165.00 (160.00, 170.00)
Weight (Kg)	71.76 ± 1.43
BMI (kg/m^2^)	25.94 ± 0.45
Systolic blood pressure (mmHg)	125.00 (114.00, 142.00)
Diastolic blood pressure (mmHg)	75.00 (69.00, 85.00)

The data of age, body weight and BMI of the patients accord with normal distribution and are expressed by mean ± SD. While the data of height, systolic blood pressure and diastolic blood pressure belong to skewed distribution and are expressed by median (interquartile range).

### 3.2 Changes of thyroid function and lipid profiles after THW

**Table 2** shows the comparisons of parameter between euthyroidism and hypothyroidism in DTC patients. DTC patients received total thyroidectomy demonstrated significantly higher TSH levels and lower thyroid hormone levels compared with those before thyroidectomy. Furthermore, significant elevations were observed in TC, TG, LDL-C, HDL-C in all DTC patients (all *P*<0.001). In addition, FBG significantly decreased after THW (*P*<0.001). Under the condition of hypothyroidism, the level of uric acid increased significantly (*P*<0.001).

**Table 2:** Parameters in different thyroid states.

	Euthyroidism	Hypothyroidism	*P-*Value
FT3 (pmol/L)	5.06 ± 0.72	2.07 ± 0.63	*P*<0.001
FT4 (pmol/L)	17.62 ± 2.77	4.36 (3.14, 5.42)	*P*<0.001
TSH (µIU/mL)	1.81 (1.34, 2.49)	61.80 ± 18.37	*P*<0.001
TC (mmol/L)	4.48 ± 0.88	5.48 ± 1.13	*P<0.001*
TG (mmol/L)	1.25 (0.90, 1.63)	1.77 (1.13, 2.51)	*P<0.001*
LDL-C (mmol/L)	2.65 ± 0.77	3.23 ± 0.93	*P<0.001*
HDL-C (mmol/L)	1.11 (0.96, 1.36)	1.24 (1.05, 1.46)	*P<0.001*
UA (mmol/L)	276 (235.00, 328.00)	309 (260.00, 362.00)	*P<0.001*
FBG (mmol/L)	5.22 (4.74, 5.80)	4.75 (4.41, 5.27)	*P<0.001*

The data that accord with normal distribution and are expressed by mean ± SD. While the data that belong to skewed distribution and are expressed by median (interquartile range).

### 3.3 Serum total cholesterol levels after THW in patients with different metabolic syndrome components before thyroidectomy

Compared with patients with normal preoperative TC, DTC patients with preoperative dyslipidemia had significantly higher total cholesterol (TC≥5.2mmol/l) after THW (*P*<0.001). However, the proportion of other metabolic syndrome components, such as hypertension, diabetes and overweight, had no effect on TC levels after THW.

### 3.4 Comparison of the prevalence of dyslipidemia between euthyroidism and hypothyroidism

**Figure 1:**
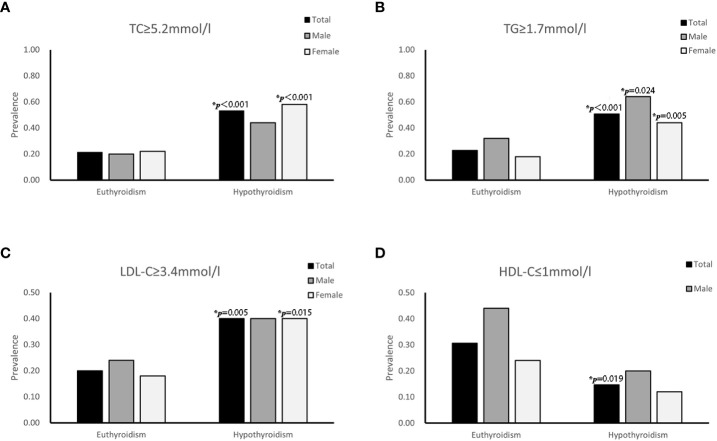
Serial changes in the proportion of patients with dyslipidemia associated with euthyroidism and hypothyroidism in patients with DTC who received radioactive iodine ablation following total thyroidectomy. Prevalence of **(A)** hypercholesteremia (TC≥5.2mmol/l), **(B)** hypertriglyceridemia (TG≥1.7mmol/l), **(C)** high low-density lipoprotein-cholesterol (LDL-C≥3.4mmol/l) and **(D)** high-density lipoprotein-cholesterol (HDL-C<1mmol/l).

Totally, compared with euthyroidism, the proportion of patients with hypertriglyceridemia, hypercholesterolemia and high LDL-C increased significantly when those patients developed hypothyroidism (**Figures 1A–C**). And the incidence of low HDL-C was reduced (*P=*0.019, **Figure 1D**). In addition, for female patients, the incidences of hypercholesteremia, hypertriglyceridemia and high LDL-C in hypothyroidism were prominently higher than that in euthyroidism (58.00% vs. 22.00%, *P*<0.001; 44.00% vs. 18.00%, *P*=0.005; 40.00% vs. 18.00%, *P*=0.015 respectively). However, in male patients, only the prevalence of hypertriglyceridemia in hypothyroidism was significantly higher than that in euthyroidism (64.00% vs. 32.00%, *P*=0.024).

### 3.5 Blood lipid levels at different TSH subgroups

The TSH levels of 23 patients (30.66%) were ≥70µIU/mL, 29 (38.67%) were at 50-70µIU/mL, 23 (30.66%) were at 30-50µIU/mL. **Table 3** summarizes the levels of blood lipids stratified by TSH group. The higher TSH levels, the significantly higher blood lipids levels. But there was no significant difference in blood lipid levels among groups with different TSH levels (*P* value: TC 0.625; TG 0.707; LDL-C 0.559; HDL-C 0.556; respectively)

**Table 3:** Serum lipid levels with different TSH subgroups.

Variables	TSH (µIU/mL)			*P* value
	30-50 (n=23)	50-70 (n=29)	≥70 (n=23)	
TC(mmol/L)	5.34 ± 1.20	5.45 ± 0.99	5.66 ± 1.25	0.625
TG(mmol/L)	1.80 ± 0.78	2.03 ± 1.09	2.03 ± 1.36	0.707
LDL-C(mmol/L)	3.16 ± 1.11	3.14 ± 0.70	3.40 ± 1.01	0.559
HDL-C(mmol/L)	1.29 ± 0.28	1.26 ± 0.32	1.35 ± 0.34	0.556

The data are expressed by mean ± SD.

### 3.6 Correlations between thyroid hormones and changes of lipids before and after THW

As shown in **Table 4**, there were negative significant correlations between FT3 levels and the changes from the euthyroidism to the hypothyroidism in TC, TG and HDL-C (*P*=0.03; *P*=0.006; *P*=0.042 respectively), and a significant positive correlation between FT4 and HDL-C (*P*=0.027) were identified in females. However, the differences were not significant in males. Meanwhile, there was no significant correlations between TSH levels and the changes from euthyroidism to hypothyroidism in serum lipid levels.

**Table 4:** Correlation between thyroid function in the hypothyroid state and changes in serum lipids.

	ΔTG_E→H_	ΔTC_E→H_	ΔLDL-C_E→H_	ΔHDL-C_E→H_
Male (n=25)				
TSH_H_	r=-0.25	r=0.12	r=0.07	r=0.23
FT3_H_	r=-0.04	r=-0.28	r=-0.32	r=-0.36
FT4_H_	r=-0.22	r=-0.34	r=-0.32	r=-0.26
Female(n=50)				
TSH_H_	r=-0.04	r=0.07	r=0.17	r=0.06
FT3_H_	r=-0.39^b^	r=-0.31^a^	r=-0.08	r=-0.29^a^
FT4_H_	r=-0.14	r=-0.25	r=-0.21	r=-0.32^a^

TSH_H_, FT4_H_, FT3_H_: TSH, FT4, FT3 level in the hypothyroid state, respectively; ΔTC_E→H_, ΔLDL-C_E→H_, ΔHDL-C_E→H_, ΔTG_E→H_: changes in serum TC, LDL-C, HDL-C, TG levels, respectively, in the transition from euthyroidism to hypothyroidism. ^a^P<0.05 or ^b^P<0.01.

## 4 Discussion

The routine treatment of DCT includes thyroidectomy, thyroid hormone inhibiting therapy and radioactive iodine ablation (if necessary). In order to elevate the iodine uptake rate before iodine ablation, thyroxine has to been withdrawn. In the current study, DTC patients who stopped taking thyroxine after partial or total thyroidectomy developed acute thyroid hormone deficiency. At this stage, some acute changes occurred in various biochemical parameters, including dyslipidemia, electrolyte disorder and so on (8).

The most common cause of secondary dyslipidemia is thyroid dysfunction. Thyroid hormones play a crucial role in maintaining lipid balance. Because thyroid hormone is associated with tissue expression and altered activities of various regulators involved in lipid metabolism, including hepatic β-hydroxy-β-methyl-glutaric acid monoacyl coenzyme A reductase (HMGCR), sterol regulatory element binding proteins (SREBPs), LDL-C receptors, cholesteryl ester transfer protein (CETP), proprotein convertase subtilsin-kexin type 9 (PCSK-9), hepatic lipase, lipoprotein lipase, and so on (9–14). Lipids and apolipoproteins that may be affected by thyroid dysfunction include TC, LDL-C, HDL-C and TG as well as apolipoprotein A and apolipoprotein B. Management of lipoprotein cholesterol is the main means to reduce the risk of atherosclerotic cardiovascular disease (15).

The overall changes of plasma lipoproteins from normal thyroid state to hypothyroidism observed in our study are consistent with previous reports of overt hypothyroidism, with elevated levels of total cholesterol, LDL-C and TG (16). About HDL-C, our results are inconsistent with previous studies, and HDL-C can be normal, elevated or decreased in patients with hypothyroidism (17–20). The result of this study showed that HDL-C increased by 9% from normal thyroid status to hypothyroidism. This may be due to the decrease of liver lipase activity that accelerates the catabolism of high density lipoprotein in hypothyroidism (21, 22). Most of LDL-C and HDL-C are synthesized in the liver, and there is competition between them. When LDL-C synthesis increases, HDL-C production decreases. Despite the increase of HDL-C, the increase of LDL-C in hypothyroidism was not reduced by the protective function of HDL-C. Therefore, elevated HDL-C level may not prevent atherosclerosis in patients with acute short-term hypothyroidism.

Previous research confirms a gender differentiation in the relationship between hypothyroidism and the lipid profile, which is substantially influenced by age (23). In our study, the proportion of hyperlipidemia is different in patients of different genders. The important reason for this phenomenon may be sex hormones the incidence of hypercholesterolemia in females was significantly higher than that in males (58.00% vs 44.00%). The possible reason might be that estrogen can inhibit the activities of hepatic lipase and lipoprotein lipase by increasing the expression of SREBP2 (SREBP2 is a key transcription factor regulating cholesterol metabolism) in hepatocytes (24, 25), that leads to increase in cholesterol levels in female. Compared with female patients, male patients had a higher incidence of hypertriglyceridemia (64.00% vs 44.00%). The possible reason might be that testosterone strongly induces triglyceride and cholesterol ester synthesis, while testosterone promotes reverse cholesterol transport by increasing scavenger receptor class B member 1 (SR-1B) expression in the liver, that leads to increase in triglyceride levels in male (26–28).,. And we further analyzed the correlation between thyroid function and changes of blood lipids in different genders. Our results show that there is no correlation between TSH level and changes in blood lipids from euthyroid state to hypothyroidism in both male and female patients. It could be because FT3 is a key regulator of body cell and tissue metabolism. It controls gene expression in target tissues by binding to the homologous nuclear receptors (TRα and TRβ) of ligand-induced transcription factors. The increase of TSH reduces the activity of liver lipase by inhibiting FT3 (29). This may be different from other studies, probably because most of the other studies are autoimmune chronic hypothyroidism, our study is acute hypothyroidism after THW. The time of hypothyroidism in this study is relatively short. When THW happens, the changes it caused in lipid may be occur with some delay. In female patients, there was a correlation between FT3 and the changes from the euthyroid state to the hypothyroid state in TC, TG, HDL-C. The severity and duration of hypothyroidism may affect the dyslipidemia associated with hypothyroidism. In our patients, hypothyroidism was severe (TSH level≥30µIU/mL), and there was no significant difference in blood lipids among different TSH subgroups. It may be suggested that when TSH increases to a certain extent, there is no significant difference in the levels of blood lipids.

Dyslipidemia is an important risk factor for atherosclerotic cardiovascular disease. Although most dyslipidemia caused by short-term acute hypothyroidism can be reversed by levothyroxine replacement therapy. Further attention should be paid to whether the fluctuation of blood lipids during this period will have a lasting effect on the risk of atherosclerosis.

Our study has some limitations. Fist, the hypothyroidism lasts only a few weeks in our study. Whether the duration of hypothyroidism is related to the prevalence of hyperlipidemia needs further study. Second, the prevalence of DTC in females is significantly higher than that in males, the number of males in this study was relatively small.

In conclusions, the present study analyzed the correlation between thyroid function and the changes of blood lipids after THW in DTC patients. And due to THW is more and more widely used before radioactive iodine ablation as a part of postoperative management of differentiated thyroid carcinoma, it is necessary to pay attention to dyslipidemia and its long-term effects after THW, especially in patients with dyslipidemia before thyroidectomy. For such patients, blood lipid management should be strengthened during this period to reduce the adverse effects of further postoperative dyslipidemia on patients.

## Data availability statement

The original contributions presented in the study are included in the article/supplementary material. Further inquiries can be directed to the corresponding authors.

## Author contributions

All authors listed have made a substantial, direct, and intellectual contribution to the work and approved it for publication.

## References

[1] Sindhu S Mb V. A study of lipid profile in patients with subclinical hypothyroidism. J Assoc Physicians India (2022) 70(4):11–2.

[2] Brenta G Fretes O. Dyslipidemias and hypothyroidism. Pediatr Endocrinol Rev (2014) 11(4):390–9. 24988692

[3] Heima NE Eekhoff EM Oosterwerff MM Lips PT van Schoor NM Simsek S. Thyroid function and the metabolic syndrome in older persons: a population-based study. Eur J Endocrinol (2013) 168(1):59–65. doi: 10.1530/EJE-12-0375 23093697

[4] Ejaz M Kumar P Thakur M Bachani P Naz S Lal K. Comparison of lipid profile in patients with and without subclinical hypothyroidism. Cureus (2021) 13(8):e17301. doi: 10.7759/cureus.17301 34567859 PMC8451506

[5] Asranna A Taneja RS Kulshreshta B. Dyslipidemia in subclinical hypothyroidism and the effect of thyroxine on lipid profile. Indian J Endocrinol Metab (2012) 16(Suppl 2):S347–9. doi: 10.4103/2230-8210.104086 PMC3603071 23565423

[6] Khatri P Neupane A Banjade A Sapkota S Kharel S Chhetri A. Lipid profile abnormalities in newly diagnosed primary hypothyroidism in a tertiary care centre of Western Nepal: A descriptive cross-sectional study. JNMA J Nepal Med Assoc (2021) 59(240):783–6. doi: 10.31729/jnma.6809 PMC9107851 34508474

[7] Su X Peng H Chen X Wu X Wang B. Hyperlipidemia and hypothyroidism. Clin Chim Acta (2022) 527:61–70. doi: 10.1016/j.cca.2022.01.006 35038435

[8] Papadakis G Kalaitzidou S Triantafillou E Drosou A Kakava K Dogkas N. Biochemical effects of levothyroxine withdrawal in patients with differentiated thyroid cancer. Anticancer Res (2015) 35(12):6933–40. 26637919

[9] Duntas LH Brenta G. A renewed focus on the association between thyroid hormones and lipid metabolism. Front Endocrinol (Lausanne) (2018) 9:511. doi: 10.3389/fendo.2018.00511 30233497 PMC6129606

[10] Tian L Song Y Xing M Zhang W Ning G Li X. A novel role for thyroid-stimulating hormone: up-regulation of hepatic 3-hydroxy-3-methyl-glutaryl-coenzyme a reductase expression through the cyclic adenosine monophosphate/protein kinase a/cyclic adenosine monophosphate-responsive element binding protein pathway. Hepatology (2010) 52(4):1401–9. doi: 10.1002/hep.23800 20648556

[11] Lopez D Abisambra Socarrás JF Bedi M Ness GC. Activation of the hepatic LDL receptor promoter by thyroid hormone. Biochim Biophys Acta (2007) 1771(9):1216–25. doi: 10.1016/j.bbalip.2007.05.001 17572141

[12] Dong B Singh AB Fung C Kan K Liu J. CETP inhibitors downregulate hepatic LDL receptor and PCSK9 expression *in vitro* and *in vivo* through a SREBP2 dependent mechanism. Atherosclerosis (2014) 235(2):449–62. doi: 10.1016/j.atherosclerosis.2014.05.931 PMC4539152 24950000

[13] Liu H Peng D. Update on dyslipidemia in hypothyroidism: the mechanism of dyslipidemia in hypothyroidism. Endocr Connect (2022) 11(2):e210002. doi: 10.1530/EC-21-0002 35015703 PMC8859969

[14] Yildirim AM Koca AO Beyan E Dogan O Karakaya S Aksoz Z. Association of serum proprotein convertase Subtilisin/Kexin type 9 (PCSK9) level with thyroid function disorders. Eur Rev Med Pharmacol Sci (2021) 25(17):5511–7. doi: 10.26355/eurrev_202109_26662 34533801

[15] Reyes-Soffer G Ginsberg HN Berglund L Duell PB Heffron SP Kamstrup PR. Lipoprotein(a): A genetically determined, causal, and prevalent risk factor for atherosclerotic cardiovascular disease: A scientific statement from the American heart association. Arterioscler Thromb Vasc Biol (2022) 42(1):e48–60. doi: 10.1161/ATV.0000000000000147 PMC9989949 34647487

[16] Erdogan M Canataroglu A Ganidagli S Kulaksızoglu M. Metabolic syndrome prevalence in subclinic and overt hypothyroid patients and the relation among metabolic syndrome parameters. J Endocrinol Invest (2011) 34(7):488–92. doi: 10.3275/7202 20651468

[17] Jain RB. Associations between the levels of thyroid hormones and lipid/lipoprotein levels: Data from national health and nutrition examination survey 2007-2012. Environ Toxicol Pharmacol (2017) 53:133–44. doi: 10.1016/j.etap.2017.05.002 28549315

[18] Shrestha N. Thyroid dysfunction and its effect in serum lipids. J Nepal Health Res Counc (2011) 9(1):33–7. 22929710

[19] Kotwal A Cortes T Genere N Hamidi O Jasim S Newman CB. Treatment of thyroid dysfunction and serum lipids: A systematic review and meta-analysis. J Clin Endocrinol Metab (2020) 105(12):dgaa672. doi: 10.1210/clinem/dgaa672 32954428

[20] Franco M Chávez E Pérez-Méndez O. Pleiotropic effects of thyroid hormones: learning from hypothyroidism. J Thyroid Res (2011) 2011:321030. doi: 10.4061/2011/321030 21760977 PMC3134217

[21] Brenta G Berg G Miksztowicz V Lopez G Lucero D Faingold C. Atherogenic lipoproteins in subclinical hypothyroidism and their relationship with hepatic lipase activity: Response to replacement treatment with levothyroxine. Thyroid (2016) 26(3):365–72. doi: 10.1089/thy.2015.0140 26839156

[22] Kobayashi J Miyashita K Nakajima K Mabuchi H. Hepatic lipase: a comprehensive view of its role on plasma lipid and lipoprotein metabolism. J Atheroscler Thromb (2015) 22(10):1001–11. doi: 10.5551/jat.31617 26194979

[23] Tognini S Polini A Pasqualetti G Ursino S Caraccio N Ferdeghini M. Age and gender substantially influence the relationship between thyroid status and the lipoprotein profile: results from a large cross-sectional study. Thyroid (2012) 22(11):1096–103. doi: 10.1089/thy.2012.0013 23050788

[24] Dalal D Dahiya K Malhotra V Aggarwal S Malik AK Ahlawat R. A comparison of reproductive hormones and biochemical parameters in hypothyroid and euthyroid postmenopausal women. Clin Lab (2020) 66(10). doi: 10.7754/Clin.Lab.2020.200243 33073944

[25] Meng Y Zong L. Estrogen stimulates SREBP2 expression in hepatic cell lines *via* an estrogen response element in the SREBP2 promoter. Cell Mol Biol Lett (2019) 24:65. doi: 10.1186/s11658-019-0194-5 31827541 PMC6892134

[26] Movérare-Skrtic S Venken K Andersson N Lindberg MK Svensson J Swanson C. Dihydrotestosterone treatment results in obesity and altered lipid metabolism in orchidectomized mice. Obes (Silver Spring) (2006) 14(4):662–72. doi: 10.1038/oby.2006.75 16741268

[27] Ibrahim N Fairus S Zulfarina MS Naina Mohamed I. The efficacy of squalene in cardiovascular disease risk-a systematic review. Nutrients (2020) 12(2):414. doi: 10.3390/nu12020414 32033387 PMC7071298

[28] Albuquerque CP Freitas FR Martinelli AEM Lima JH Coelho RF Serrano CV Jr. Androgen deprivation therapy improves the *in vitro* capacity of high-density lipoprotein (HDL) to receive cholesterol and other lipids in patients with prostate carcinoma. Lipids Health Dis (2020) 19(1):133. doi: 10.1186/s12944-020-01305-8 32522195 PMC7285573

[29] Sinha RA You SH Zhou J Siddique MM Bay BH Zhu X. Thyroid hormone stimulates hepatic lipid catabolism *via* activation of autophagy. J Clin Invest (2012) 122(7):2428–38. doi: 10.1172/JCI60580 PMC3386813 22684107

